# Diet Quality Scores and Risk of Nasopharyngeal Carcinoma in Chinese Adults: A Case-Control Study

**DOI:** 10.3390/nu8030112

**Published:** 2016-02-25

**Authors:** Cheng Wang, Xiao-Ling Lin, Yu-Ying Fan, Yuan-Ting Liu, Xing-Lan Zhang, Yun-Kai Lu, Chun-Hua Xu, Yu-Ming Chen

**Affiliations:** 1 Guangdong Provincial Key Laboratory of Food, Nutrition and Health, School of Public Health, Sun Yat-sen University, Guangzhou 510080, China; wangch28@mail2.sysu.edu.cn; 2 Department of Gynecologic Oncology, Sun Yat-sen University Cancer Center, Guangzhou 510060, China; Linxiaol@sysucc.org.cn; 3 Sun Yat-sen University Ophthalmic Center, Guangzhou 510060, China; Fanyy@sysucc.org.cn (Y.-Y.F.); luyunkai@mail.sysu.edu.cn (Y.-K.L.); 4 Information Section, Central Hospital of Panyu District, Guangzhou 511400, China; tingty@126.com; 5 Department of Radiotherapy, Sun Yat-sen University Cancer Center, Guangzhou 510060, China; Zhangxingl@sysucc.org.cn; 6 Clinical laboratory section of the office outpatient Department public security board, Guangdong 510050, China; ladychun@126.com

**Keywords:** nasopharyngeal carcinoma, diet quality, dietary pattern, case-control study, Chinese, adults

## Abstract

Many studies show that dietary factors may affect the risk of nasopharyngeal carcinoma (NPC). We examined the association between overall diet quality and NPC risk in a Chinese population. This case-control study included 600 NPC patients and 600 matched controls between 2009 and 2011 in Guangzhou, China. Habitual dietary intake and various covariates were assessed via face-to-face interviews. Diet quality scores were calculated according to the Healthy Eating Index-2005 (HEI-2005), the alternate Healthy Eating Index (aHEI), the Diet Quality Index-International (DQI-I), and the alternate Mediterranean Diet Score (aMed). After adjustment for various lifestyle and dietary factors, greater diet quality scores on the HEI-2005, aHEI, and DQI-I—but not on the aMed—showed a significant association with a lower risk of NPC (*p*-trends, <0.001–0.001). The odds ratios (95% confidence interval) comparing the extreme quartiles of the three significant scores were 0.47 (0.32–0.68) (HEI-2005), 0.48 (0.33–0.70) (aHEI), and 0.43 (0.30–0.62) (DQI-I). In gender-stratified analyses, the favorable association remained significant in men but not in women. We found that adherence to the predefined dietary patterns represented by the HEI-2005, aHEI, and DQI-I scales predicted a lower risk of NPC in adults from south China, especially in men.

## 1. Introduction

Nasopharyngeal carcinoma (NPC), which originates in the epithelial cells lining the nasopharynx, is a rare type of neoplasm worldwide. The Global Cancer estimation project for 2012 indicated an age-standardized incidence of 1.2 and mortality rate of 0.7 per 10^5^ [1]. However, much higher incidences have been observed in southern China, especially in the province of Guangdong (incidence, 10.5 × 10^−5^; mortality rate, 5.3 × 10^−5^) in 2011 [2]. More efforts are needed to explore the risk factors of NPC to allow more effective prevention in the Guangdong population.

NPC is believed to result from a combination of genetic susceptibility, Epstein–Barr virus infection, and a variety of environmental factors (e.g., carcinogens and dietary factors) [3,4]. An increasing body of evidence has shown that dietary factors may have an enigmatic effect on the initiation and promotion of NPC [4,5,6]. Studies have consistently reported a positive association between the risk of NPC and the consumption of salt-preserved fish [7,8], salted vegetables, and preserved meat [9,10], and favorable associations have been observed between the intake of fresh fruit and vegetables and the risk of NPC in Chinese adults [4,11]. However, data on the role of overall dietary quality in the risk of NPC are scarce.

A larger number of scores have been developed to assess overall dietary quality according to various dietary guidelines, such as the Healthy Eating Index-2005 (HEI-2005) [12], the alternate Healthy Eating Index (aHEI) [13], the Diet Quality Index-International (DQI-I) [14], and other beneficial dietary patterns (e.g., the alternate Mediterranean Diet (aMed) [15]). Many studies have shown associations between higher overall diet scores and a lower risk of various cancers, including head and neck cancer [16], gastric cancer [17], esophageal cancer [18], rectal cancer [19], and breast cancer [20]. Moreover, different dietary scales have different predictive values on the risk of individual cancers. However, to our knowledge, no study has yet reported associations between overall diet quality scores and the risk of NPC.

This study was performed to determine the association between four widely used diet quality scores (HEI-2005, aHEI, DQI-I, and aMed) and the risk of NPC and to assess which scores may best predict the risk of NPC in a high-incidence population in southern China.

## 2. Methods 

### 2.1. Study Population 

This matched case-control study was conducted between July 2009 and March 2011 in Guangzhou, Guangdong Province, China. The design of the study was described previously [11]. The patients and control subjects were matched in a 1:1 ratio by gender, age (±3 years), and household type (urban *vs.* rural in the past 10 years). The detailed inclusion and exclusion criteria for patients and control subjects are shown in Supplemental Table S1. The ethics committee of the School of Public Health of Sun Yat-sen University approved the study, and written informed consent was obtained from all participants.

### 2.2. Data Collection

Face-to-face interviews were conducted by experienced interviewers with relevant medical knowledge using a structured questionnaire that included information on (1) sociodemographic characteristics; (2) occupational and domestic exposure to toxic substances; (3) lifestyle habits (e.g., smoking and alcohol consumption); (4) habitual dietary consumption over the year before diagnosis (patients) or before the interview (control subjects); (5) all physical activities, including occupational and leisure activities, over the past month before admission to hospital (or interview); and (6) history of chronic diseases. Each interviewer completed an equal proportion of questionnaire interviews between the patient and control groups.

### 2.3. Assessment of Dietary Intake

A semiquantitative 78-item food frequency questionnaire (FFQ) was used to assess dietary consumption [11,21]. The mean intake of food per day, week, month, or year was reported for each food item on the FFQ. For seasonal foods, the participants were asked to report how many months of the year they consumed each item. Food photographs were provided as visual aids to assess portion sizes. The average daily intake of nutrients and total energy were calculated according to the Chinese Food Composition Table, 2002 [22]. We adjusted grams per day to servings per day according to the serving size definitions of the U.S. Food Guide Pyramid (2005) and Chinese dietary guidelines (2007) when appropriate [23].

### 2.4. Calculation of Diet Quality Scores

We calculated the diet quality scores of HEI-2005, aHEI, DQI-I, and aMed on the basis of the data from the FFQ interview. The calculations for the HEI-2005, aHEI, and DQI-I were mainly based on the distribution of intake of foods, nutrients, energy, and/or ratios of the relevant items, or the median values for aMed, as described in detail in previous reports [24].

The 100-point HEI-2005 scale includes 14 items [12]. Fruits, vegetables, grains, milk, meat and beans, and oils were measured as servings per 1000 kcal, and solid fat, alcohol, and added sugar were expressed as the percentage of total calories. For sodium, we only asked whether the subject’s taste was light, moderate, or heavy, and each corresponding choice was valued 0, 5, or 10 points, respectively.

The nine-item aHEI score was developed by McCullough *et al.* [13] based on foods and nutrients (e.g., nut and soy protein, cereal fiber, *trans*fats, and the ratio of polyunsaturated to saturated fat) that are associated with a risk of chronic disease. Each component is worth 0 to 10 points except for the multivitamin item, which was either 2.5 or 7.5. We excluded *trans*fats because they are consumed at a very low level in the middle-aged and elderly Chinese population [25]. Therefore, the aHEI scores contained eight components for a total of 77.5 points in our study.

The DQI-I score was calculated on the basis of the methods developed by Kim *et al.* [14]. Briefly, the DQI-I assesses four categories (variety, adequacy, moderation, and overall balance) and contains six food items and 11 nutrient items. The maximum possible score for each item ranges from 4 to 15 points, for a total score of 100 points.

The aMed score was modified by Fung *et al.* [15] from the original Mediterranean diet score [26] to evaluate nine components with a maximum score of 9. One point for each item is assigned to subjects with high intake of whole grains, vegetables, fruit, legumes, nuts, fish, ratio of monounsaturated to saturated fat, a moderate intake of alcohol, and a low intake of red and processed meats, respectively, based upon the sex-specific median in the control subjects.

### 2.5. Statistical Analysis

Common characteristics between the patients and the control subjects were compared using paired *t*-tests for continuous variables and paired chi-square tests for categorical variables. Logarithmic transformation was used for energy intake, and square root transformation was applied to the other dietary factors. Dietary intakes were adjusted for total energy intake using the residual method. The diet quality scores on the HEI-2005, aHEI, DQI-I, and aMED were categorized into four quartiles (Q1 to Q4) on the basis of the sex-specific quartile cutoffs among the control subjects. Odds ratios and 95% confidence intervals (CIs) for NPC were estimated with the use of univariate and multivariate conditional logistic regression models for each score using the lowest quartile (Q1) as the referent. *p* values for the between quartiles were also calculated using quartile 2 and 3 as the referent, respectively. The *p* values for linear trends were estimated by modeling the quartiles of diet quality scores as continuous variables. In the multivariate model, we adjusted for age, body mass index, occupation, marital status, education level, household income, current smoking status, current drinking status, exposure to potential toxic substances, history of chronic rhinitis, physical activity, energy intake, intake of preserved vegetables and animal food, and multivitamin supplements, except for the aHEI score analysis. Stratified analyses were performed by gender. The interaction significance was evaluated with the Wald *χ*^2^ test with a multiplicative interaction term (α = 0.05 divided by the number of tests). Statistical significance was inferred for two-tailed *p* values of less than 0.05. All statistical analyses were performed with SPSS software (version 17.0, SPSS, Chicago, IL, USA).

## 3. Results

We screened 653 eligible patients from all 851 inpatients in the hospital during the study period. We excluded those with an incomplete questionnaire (20 patients), those who refused to participate (31 patients), and those who had implausible daily energy intakes (700 to 4200 kcal for men and 500 to 3500 kcal for women) (2 patients). The final analysis included 600 patients and 600 matched control subjects. 

The patients with NPC and the control subjects had similar distributions of age, gender, and household type. There were three times as many men (74.7%) as women. The patients with NPC were more likely than the control subjects to have a history of chronic rhinitis (*p* < 0.001), a higher body mass index (*p* < 0.002), less intense occupational activity (*p* < 0.001) and physical activity (*p* = 0.013), and lower total energy intake (*p* = 0.029). No significant differences were observed for current drinking status, current smoking status, and exposure to toxic substances (Table 1).

The median scores for patients and control subjects were 71 (range, 35 to 86) and 72 (32 to 87) for the HEI-2005, 39.5 (13.5 to 62.5) and 41.5 (11.5 to 62.5) for the aHEI, 52 (22 to 74) and 54 (27 to 74) for the DQI-I, and 4 (0 to 8) and 4 (0 to 9) for the aMed. The diet quality scores all had significant correlations (all *p* < 0.01) to each other (Supplemental Table S2).

The univariate analyses showed that subjects with higher scores on the HEI-2005, aHEI, and DQI-I—but not the aMed—had a significantly lower risk of NPC (*p* trends, <0.001 to 0.006). Similar associations remained after adjustment for multiple covariates (*p* trends, <0.001 to 0.001) (Table 2). The odds ratios (95% CI) of NPC risk for the extreme quartiles (Q4 *vs.* Q1) of the diet quality scores were 0.47 (0.32 to 0.68) for the HEI-2005 score, 0.48 (0.33 to 0.70) for the aHEI score, and 0.43 (0.30 to 0.62) for the DQI-I score, respectively (Table 2).

In gender-stratified analyses, significant associations between the HEI-2005, aHEI, and DQI-I scores, and the risk of NPC were observed only in men but not in women. The corresponding odds ratios (Q4 *vs.* Q1) ranged from 0.36 to 0.40 in men and 0.55 to 0.72 in women for the three scores (*p* interactions: 0.048 to 0.212) (Table 3).

## 4. Discussion

This case-control study with 600 patients and 600 control subjects showed that three diet quality scores (HEI-2005, aHEI, and DQI-I) had approximate favorable associations with the risk of NPC, but the aMed score had no such association. These results suggest that adherence to these three indices could play a role in the prevention of NPC. Although these three scores had similar associations with the risk of NPC, public health messages should pay more attention to the aHEI score for the prediction of NPC because it has fewer scoring items and the simplest scoring method.

Up to date, no study has yet reported the relationship between diet quality scores and the risk of NPC. However, significant inverse associations have been shown between diet quality scores and the risks of many other cancers in most [18,19,27,28,29,30], but not all [31,32], previous studies. In the National Institutes of Health–AARP Diet and Health Study with 537,218 men and women (followed-up for 10.5 years), the consumption of a diet with a high HEI-2005 and aHEI score may reduce the risk of pancreatic [27], prostate [28], esophageal [18], and colorectal [19] cancers, with multivariable adjusted hazard ratios ranging from 0.51 to 0.92 when comparing the highest and lowest quintiles. Data from the Health Professional’s Follow-up Study and Nurses’ Health Study showed that the HEI-2005 and aHEI-2010, which are strongly correlated with the aHEI, had an inverse association with the overall risk of cancer among women and men (*p* trend, 0.003 and 0.001, respectively) [29]. Similar findings were seen in a recent systematic review and meta-analysis of cohort studies, in which diets of the highest quality, as assessed by the HEI, aHEI, and DASH (Dietary approach to stop hypertension) score, resulted in a significant reduction in the incidence and mortality risk of cancer (relative risk, 0.85; 95% CI, 0.82 to 0.88) [30]. Few studies have examined the association between cancer risk and the DQI-I, although a higher DQI-I score was shown to be associated with a reduced risk of other chronic diseases, such as non-alcoholic fatty liver disease and diabetes, that may promote cancer risk [33,34]. We found that subjects with scores in the highest (*vs.* lowest) quartile of the HEI-2005, aHEI, and DQI-I had 52% to 57% lower risks of NPC (*p* trend, <0.001 to 0.001); these findings are consistent with those in other cancers in previous studies. Our results may provide new evidence for overall dietary quality in the prevention of NPC. Further studies are needed to address whether the favorable association is site specific, as null associations are likely to be observed in some hormone-related cancers, such as endometrial cancer [31], ovarian cancer [32] and estrogen-receptor–positive breast cancer [20].

The beneficial effects of high-quality dietary scores may reflect the synergistic effects of diverse foods and numerous nutrients characterized in higher vegetables, fruit, total grains, and nuts consumption. Previous studies suggested that greater consumption of vegetables and fruit may lower the risk of NPC [11,35]. In the individual component analyses, we found significant inverse associations between vegetables, fruit, fiber, and vitamin C components and the incidence of NPC (Supplemental Table S3). Many ingredients of vegetables and fruit may contribute to a reduction in NPC risk, including dietary fiber, antioxidants, and other phytochemicals [36]. Antioxidants, such as vitamin A, C, and E and carotenoids, contribute to the prevention of nasopharyngeal oncogenesis via regulation of the progress of cell differentiation and proliferation, reduction of oxidative DNA fragmentation in nasal epithelial cells, and inhibition of the early antigen expression of the Epstein–Barr virus [37,38,39]. Moreover, inflammation is a major feature in carcinogenesis, and the overexpression of inflammation cytokine has been a promoter for NPC progression [40]. Higher diet quality scores have been related to lower concentrations of inflammatory biomarkers, which may help prevent the development of NPC [41].

Although the various dietary quality scores all emphasized greater intake of fruits and vegetables and less intake of saturated fat, only the aMed showed no significant association with NPC in our study, which is inconsistent with several reports in which the aMed showed an inverse relationship to the risk of other cancers [26,42,43]. A partial reason for the null association may be the differences in the numbers of score items and scoring criteria. The aMed has a much narrower possible score range, and its significant components had relatively lower weight (Supplemental Table S3) compared to those in the HEI-2005, aHEI, and DQI-I. Thus, the aMed score may not be able to finely discriminate dietary healthfulness in terms of NPC risk in our population. In addition, Davis *et al.* [44] recently found that the average nutrients and bioactive contents of the diet was relatively consistent in the food groups among previous studies in which the Mediterranean diet showed a positive effect on chronic disease. More studies are needed to confirm our findings with regard to NPC.

In gender-stratified analyses, the favorable association between the HEI-2005, aHEI, and DQI-I scores and NPC remained significant in men but not in women, although no significant gender interactions were observed (*p* interaction, 0.055 to 0.212, >0.05/4 tests). These differences might be partly a result of genetic variations in NPC susceptibility, with almost twofold incidence in men than in women and stronger acceleration of dietary risk factors on the relevant biomarkers of inflammation in men [45,46]. Posteriorly, the relatively small sample size for women (case-control pairs, men *vs.* women: 448 *vs.* 152) might contribute to the gender differences.

Our study has several strengths. To our knowledge, this was the first study to systematically test the associations between diet quality scores derived from various dietary guidelines and the risk of NPC using a well-constructed FFQ with satisfactory reproducibility and validity [21]. The interviewers were well-trained, and each interviewer surveyed the same proportion of patients and control subjects. A 1:1 matched study was designed to minimize the influence of age, gender, and household types. In addition, adjustments were made in the multivariate analysis for a number of potential covariates, including socioeconomic status, body mass index, and some dietary factors (such as intake of preserved vegetables and animal foods).

This study also has several limitations that should be considered. First, inverse causality could not be fully excluded in a case-control study because the diet information was collected after the diagnosis of NPC. To minimize this possibility, only new cases (within three months of diagnosis) were included in our study, and both the patients and the control subjects were confined to those without a substantial change in dietary habits over the previous five years. Moreover, patients were more likely to change their dietary habits in a favorable direction, which might underestimate the protective effect of dietary patterns. Second, recall bias of one’s habitual diet is unavoidable in retrospective data collection, but differential report bias between patients and control subjects was unlikely to be present in this study because NPC is considered to be a major genetic and virus-related cancer, and there was little public awareness of a potential dietary influence except for salted or preserved foods. Finally, the patients with NPC and the control subjects were recruited from two different hospitals, which might have resulted in a selection bias. However, both hospitals were top-ranking specialized institutions with comparable reputations and coverage areas. The distributions of the major demographic characteristics (for example, age and sex) in our study matched those in a population-based epidemiological survey of NPC [47].

## 5. Conclusions

In summary, our results first suggest that the dietary patterns represented by the HEI-2005, AHEI and DQI-I scores predict a lower risk of NPC in adults in southern China, especially in men. Our findings provide evidence for adherence to the general dietary guidelines for the prevention of NPC. We found no association between the aMED score and NPC, which differs from studies about many other chronic diseases. Further prospective studies are merited to confirm our findings.

## Figures and Tables

**Table 1:** Participants’ characteristics in nasopharyngeal carcinoma cases and controls *^a^*.

	Cases (*n* = 600)	Controls (*n* = 600)	*p*-Value
Age, year	47.4 ± 9.0	47.4 ± 9.0	0.992
Gender (Male/Female)	448/152	448/152	
Body mass index, kg/m^2^	23.2 ± 3.1	22.7 ± 2.8	0.002
Marital status			0.007
Married	590 (98.3)	574 (95.7)	
Single	10 (1.7)	26 (4.3)	
Household type, *n* (%)			
Urban	399 (66.5)	399 (66.5)	
Rural	201 (33.5)	201 (33.5)	
Education, *n* (%)			0.001
Primary school or below	109 (18.2)	130 (21.7)	
Secondary school	190 (31.7)	229 (38.2)	
High school	170 (28.3)	158 (26.3)	
College or above	131 (21.8)	83 (13.8)	
Family monthly income, Yuan/person			0.007
<1500	256 (42.7)	304 (50.7)	
1500–3000	158 (26.3)	153 (25.5)	
>3000	186 (31.0)	143 (23.8)	
Occupation, *n* (%)			<0.001
Light intensity of activity	228 (38.0)	192 (32.0)	
Moderate intensity of activity	188 (31.3)	158 (26.3)	
Heavy intensity of activity	184 (30.7)	250 (41.7)	
Chronic rhinitis history, *n* (%)	164 (27.3)	108 (18.0)	<0.001
Exposure to toxic substances, *n* (%) *^b^*	274 (45.7)	269 (44.8)	0.814
Current drinking, *n* (%) *^c^*	132 (22.0)	121 (20.2)	0.489
Current smoking, *n* (%) *^d^*	294 (49.0)	277 (46.2)	0.343
Physical activities, MET h/day *^e^*	38.2 ± 8.9	39.7 ± 12.2	0.013
Dietary energy intake, kcal/day	1873 ± 600	1953 ± 642	0.029
Multivitamin use, *n* (%)	32 (5.3)	35 (5.8)	0.711

*^a^* Continuous values are described by means ± SD. Categorical variables are described by numbers (%); *^b^* Exposure to potential toxic substances or detrimental environment included exposuring to one of the following factors over one year: heat, organic solvents, pesticides, heavy metals, smoke from burning incense, anti-mosquito coils, new furniture or decoration and radiation; *^c^* Current drinking was defined as having had alcohol drinks at least once weekly for at least six consecutive months; *^d^* Current smoking was defined as having smoked at least one cigarette daily for at least six consecutive months; *^e^* Physical activities included daily occupational, leisure-time and household-chores, evaluated by metabolic equivalent (MET) hours per day.

**Table 2:** ORs (95% CIs) of nasopharyngeal carcinoma for quartiles of diet-quality scores (*n* = 600 pairs) *^a^*.

	ORs (95% CI) by Quartiles of Each Scores				*p*-Trend
	Q1	Q2	Q3	Q4 (Highest)	
HEI-2005					
*N* (case/control)	189/175	165/146	168/144	78/135	
Score *^b^*	62	69	75	80	
Crude OR	1.00	1.02 (0.76,1.38)	1.01 (0.75,1.36)	0.54 (0.38,0.77) **^,##,&&^	0.006
Adjusted OR *^c^*	1.00	0.95 (0.69,1.31)	0.90 (0.65,1.24)	0.47 (0.32,0.68) **^,##,&&^	0.001
aHEI					
*N* (case/control)	196/158	180/149	128/153	96/140	
Score	31.5	39.5	44.5	50.5	
Crude OR	1.00	0.93 (0.69,1.24)	0.64 (0.46,0.89) **^,#^	0.54 (0.39,0.76) **^,##^	<0.001
Adjusted OR	1.00	0.93 (0.67,1.28)	0.50 (0.34,0.73) **^,#^	0.48 (0.33,0.70) **^,#^	<0.001
DQI-I					
*N* (case/control)	229/164	115/151	163/142	93/143	
Score	44	52	57	63	
Crude OR	1.00	0.53 (0.38,0.73) **	0.81 (0.60,1.11) ^#^	0.45 (0.32,0.64) **^,&&^	<0.001
Adjusted OR	1.00	0.45 (0.31,0.64) **	0.75 (0.53,1.05) ^#^	0.43 (0.30,0.62) **^,&&^	<0.001
aMed					
*N* (case/control)	137/136	122/103	185/181	156/180	
Score	2	3	4	6	
Crude OR	1.00	1.17 (0.82,1.66)	1.01 (0.73,1.38)	0.86 (0.62,1.18)	0.240
Adjusted OR	1.00	1.06 (0.72,1.56)	0.98 (0.69,1.37)	0.85 (0.59,1.22)	0.319

*^a^* Abbreviations: aHEI: alternate Healthy Eating Index; aMed: alternate Mediterranean Diet Score; DASH: Dietary approach to stop hypertension; DQI-I: Diet Quality Index–International; HEI-2005: Healthy Eating Index—2005; ORs (95% CI): Odds ratios (95% confidence interval); **: Compared with Quartile 1, *p* < 0.01; ^#,##^: Compared with Quartile 2, *p* < 0.05, *p* < 0.01; ^&&^: Compared with Quartile 3, *p* < 0.01; *^b^* Median score in controls; *^c^* Crude and adjusted ORs (95% CI) from conditional logistic regression models. For the adjusted ORs, covariates include age, body mass index, occupation, marital status, educational level, household income, current smoking, current drinking, exposure to potential toxic substances, multivitamin supplements, chronic rhinitis history, physical activity, daily energy intake, preserved vegetables and animal food, and multivitamin supplements except for the aHEI score analysis.

**Table 3:** Adjusted ORs (95% CIs) of nasopharyngeal carcinoma for quartiles of diet-quality scores by gender ^*a*^.

	ORs (95% CI) *^b^* by Quartiles of Each Score				*p*-Trend	*p*-Inter-Action
	Q1	Q2	Q3	Q4 (Highest)		
HEI-2005						
Gender						0.055
Men	1.00	0.90 (0.61,1.33)	0.79 (0.53,1.19)	0.40 (0.25,0.65) **^,##,&^	0.001	
Women	1.00	1.13 (0.56,2.28)	1.54 (0.83,2.86)	0.71 (0.32,1.57)	0.934	
aHEI						
Gender						0.212
Men	1.00	0.90 (0.61,1.32)	0.47 (0.31,0.74) **	0.40 (0.26,0.62) **	<0.001	
Women	1.00	1.07 (0.58,1.97)	0.66 (0.30,1.42)	0.72 (0.35,1.47)	0.221	
DQI-I						
Gender						0.048
Men	1.00	0.41 (0.27,0.64) **	0.61 (0.41,0.92) *^,#^	0.36 (0.23,0.55) **^,&&^	<0.001	
Women	1.00	0.55 (0.26,1.16)	1.25 (0.62,2.49)	0.55 (0.25,1.22)	0.644	
aMed						
Gender						0.199
Men	1.00	1.10 (0.70,1.72)	0.99 (0.67,1.46)	0.74 (0.48,1.15)	0.178	
Women	1.00	1.64 (0.74,3.63)	0.79 (0.35,1.76)	1.26 (0.65,2.44)	0.797	

*^a^* Abbreviations: see Table 2; *n*: men 448 pairs, women 152 pairs; *^,^**: Compared with Quartile 1, *p* < 0.05, *p* < 0.01; ^#,##^: Compared with Quartile 2, *p* < 0.05, *p* < 0.01; ^&,&&^: Compared with Quartile 3, *p* < 0.05, *p* < 0.01; *^b^* Odds ratios (95% CI): from multivariate conditional logistic regression models after adjustments for the covariates as indicated in Table 2. α for interactions = 0.05/4 tests = 0.0125.

## Supplementary Materials

The following are available online at http://www.mdpi.com/2072-6643/8/3/112/s1, Table S1: Inclusion and exclusion criteria for NPC cases and controls, Table S2. Spearman’s rank correlation coefficients for NPC cases (*n* = 600) and controls (*n* = 600) among total summary scores for the HEI-2005, aHEI, DQI-I, and aMed scores, Table S3. Odds ratio (95% CIs) of nasopharyngeal carcinoma for the highest (*vs.* lowest) quartile of the individual components of the selected diet-quality scores.

## Abbreviations

aHEI: alternate Healthy Eating Index; aMed: alternate Mediterranean Diet Score; DASH: Dietary approach to stop hypertension; DQI-I: Diet Quality Index–International; HEI-2005: Healthy Eating Index–2005; NPC: nasopharyngeal carcinoma.
