# Supplementary material for: Diet Quality Scores and Risk of Nasopharyngeal Carcinoma in Chinese Adults: A Case-Control Study
Source: Nutrients. 2016 Feb 25;8(3):112. doi: 10.3390/nu8030112 (PMC4808842; doi:10.3390/nu8030112)
Supplement: Supplementary file 1 [file nutrients-08-00112-s001.docx]

Supplementary Materials: Diet Quality Scores and Risk of Nasopharyngeal Carcinoma in Chinese Adults: A Case-Control Study

Cheng Wang, Xiao-Ling Lin, Yu-Ying Fan, Yuan-Ting Liu, Xing-Lan Zhang, Yun-Kai Lu,
Chun-Hua Xu and Yu-Ming Chen

**Table S1.** Inclusion and exclusion criteria for NPC cases and controls.

|  | **Cases** | **Controls** |
| --- | --- | --- |
| Inclusion criteria | Residents of Guangdong Province for more than 10 years | |
|  | Inpatients at the Sun Yat-sen University Cancer Center with histological diagnoses of NPC up to 3 months prior to interviews | Inpatients hospitalized in the  Sun Yat-sen University Ophthalmic Center within 1 week |
| Exclusion criteria | With a family history of NPC within  3 generations | With diseases affect routine activities *^a^* |
|  | Reported substantial changes in dietary habit in the last 5 years | |
|  | Chronic diseases *^b^* that might change dietary habit | |
|  | Could not speak Mandarin/Cantonese | |
|  | Reported implausible energy intake *^c^* and had insufficient data | |

*^a^* Diseases included any cancer, cataracts, or poor vision; *^b^* Chronic diseases included diabetes, stroke, coronary disease, other malignant cancers, cognitive disorder, liver cirrhosis, renal failure, thyroid disorder and chronic diarrhea; *^c^* Implausible energy intake range: <700 or >4200 kcal per day for males; <500 or >3500 kcal per day for females.

**Table S2.** Spearman’s rank correlation coefficients for NPC cases (*n* = 600) and controls (*n* = 600) among total summary scores for the HEI-2005, aHEI, DQI-I, and aMed scores.

|  | **HEI-2005** | | **aHEI** | | **DQI-I** | | **aMed** | |
| --- | --- | --- | --- | --- | --- | --- | --- | --- |
|  | **Cases** | **Controls** | **Cases** | **Controls** | **Cases** | **Controls** | **Cases** | **Controls** |
| HEI-2005 | 1.000 | 1.000 | 0.609 | 0.595 | 0.755 | 0.689 | 0.525 | 0.494 |
| aHEI |  |  | 1.000 | 1.000 | 0.630 | 0.649 | 0.704 | 0.750 |
| DQI-I |  |  |  |  | 1.000 | 1.000 | 0.522 | 0.568 |
| aMed |  |  |  |  |  |  | 1.000 | 1.000 |

HEI-2005 Healthy Eating Index-2005, aHEI alternate Healthy Eating Index, DQI-I Diet Quality Index-International, aMed alternate Mediterranean Diet Score. All *p* < 0.01.

**Table S3.** Odds ratio (95% CIs) of nasopharyngeal carcinoma for the highest (*vs*. lowest) quartile of the individual components of the selected diet-quality scores *^a^*.

| **Components** | **Median Score, Cases/Controls** | **ORs (95% CI)** | ***p* Value** |
| --- | --- | --- | --- |
| **HEI-2005** |  |  |  |
| Total fruit (included juice) | 2/2 | 0.96 (0.91, 1.01) | 0.115 |
| Whole fruit (except juice) | 4/4 | 0.96 (0.91, 1.01) | 0.104 |
| Total Vegetables (included potatoes) | 4/4 | 0.90 (0.85, 0.96) | 0.002 |
| Dark Green and Orange Vegetables and Legumes | 5/5 | 0.88 (0.82, 0.95) | 0.001 |
| Total Grains | 5/5 | - | - |
| Whole Grains | 5/5 | 0.99 (0.95, 1.03) | 0.620 |
| Milk | 0/0 | 0.85 (0.27, 2.67) | 0.784 |
| Meat and Beans | 10/10 | 0.97 (0.89, 1.07) | 0.562 |
| Oils | 10/10 | 1.06 (0.93, 1.21) | 0.363 |
| Saturated Fat | 8/8 | 1.01 (0.98, 1.04) | 0.546 |
| Calories from solid fat, alcohol, and added sugar (SoFAAS) | 20/20 | 1.00 (0.97, 1.03) | 0.959 |
| Sodium (taste) | 3/3 | 0.98 (0.95, 1.02) | 0.355 |

**Table S3.** *Cont.*

| **Components** | **Median Score, Cases/Controls** | **ORs (95% CI)** | ***p* Value** |
| --- | --- | --- | --- |
| **aHEI** |  |  |  |
| Vegetables (except potatoes) | 7/6 | 0.95 (0.92, 0.98) | <0.001 |
| Fruit (included juice) | 3/3 | 0.97 (0.93, 0.99) | 0.029 |
| Nuts and soy protein | 10/10 | 0.99 (0.96, 1.02) | 0.463 |
| Cereal fiber | 7/6 | 0.93 (0.89, 0.97) | 0.001 |
| Ratio of white to red meat | 1/2 | 1.01 (0.99, 1.04) | 0.326 |
| Ratio of polyunsaturated to saturated fat | 10/10 | 1.02 (0.94, 1.09) | 0.695 |
| Long-term multivitamin regular use | 2.5/2.5 | 0.166 (0.00, +∞) | 0.912 |
| Alcohol | 0/0 | 1.01 (0.92, 1.10) | 0.897 |
| **DQI-I** |  |  |  |
| **Variety** |  |  |  |
| Overall food group variety (meat/poultry/fish/eggs; dairy/beans;  grain; fruit; vegetable) | 12/12 | 0.98 (0.94, 1.01) | 0.220 |
| Within-group variety for protein source  (meat, poultry, fish, dairy, beans, eggs) | 5/5 | 1.01 (0.94, 1.09) | 0.735 |
| **Adequacy** |  |  |  |
| Vegetables | 5/5 | 0.90 (0.84, 0.97) | 0.004 |
| Fruit | 3/3 | 0.96 (0.91, 1.01) | 0.135 |
| Grains | 3/3 | 1.00 (0.93, 1.07) | 0.946 |
| Fiber | 3/3 | 0.89 (0.82, 0.97) | 0.009 |
| Protein | 5/5 | 0.97 (0.80, 1.18) | 0.784 |
| Iron | 5/5 | 1.03 (0.87, 1.22) | 0.713 |
| Calcium | 3/3 | 0.93 (0.86, 1.01) | 0.085 |
| Vitamin C | 5/5 | 0.90 (0.84, 0.97) | 0.003 |
| 1. **Moderation** |  |  |  |
| Total fat | 0/0 | 1.05 (0.99, 1.11) | 0.108 |
| Saturated fat | 3/3 | 1.02 (0.98, 1.07) | 0.341 |
| Cholesterol | 3/3 | 1.01 (0.97, 1.04) | 0.779 |
| Sodium (taste) | 3/3 | 0.98 (0.95, 1.02) | 0.355 |
| Empty calorie foods (oils, alcohol, starch) | 0/0 | 0.97 (0.83, 1.13) | 0.653 |
| 1. **Overall balance** |  |  |  |
| Macronutrient ratio (carbohydrate:protein:fat) | 0/0 | 1.08 (0.78, 1.49) | 0.644 |
| Fatty acid ratio (PUFA:MUFDA:SFA) | 2/2 | 0.98 (0.90, 1.06) | 0.572 |
| **Alternate Mediterranean Diet Score (aMed)** |  |  |  |
| Whole grains | 0.5/0 | 0.98 (0.83, 1.16) | 0.982 |
| Vegetables (except potatoes) | 0.5/0 | 0.79 (0.67, 0.94) | 0.009 |
| Fruits (included juices) | 0.5/0 | 0.90 (0.76, 1.07) | 0.225 |
| Legumes | 0.5/0 | 0.87 (0.74, 1.03) | 0.106 |
| Nuts | 0.5/0 | 0.91 (0.77, 1.07) | 0.243 |
| Fish | 1/1 | 1.20 (1.00, 1.44) | 0.050 |
| Ratio of monounsaturated to saturated fat | 1/0 | 1.01 (0.86, 1.20) | 0.870 |
| Red and processed meats | 0.5/1 | 1.04 (0.88, 1.23) | 0.629 |
| Alcohol | 0/0 | 1.34 (0.89, 2.02) | 0.159 |

*^a^* Odds ratios (95% CI) were from multivariate conditional logistic regression models. Covariates includes age, body mass index, occupation, marital status, educational level, household income, current smoking, current drinking, exposure to potential toxic substances, multivitamin supplement, chronic rhinitis history, physical activity, daily energy intake, preserved vegetables and animal food, and multivitamin supplement except for the aHEI score analysis.
